# Modern Soft-Sensing Modeling Methods for Fermentation Processes

**DOI:** 10.3390/s20061771

**Published:** 2020-03-23

**Authors:** Xianglin Zhu, Khalil Ur Rehman, Bo Wang, Muhammad Shahzad

**Affiliations:** School of Electrical and Information Engineering, Jiangsu University, Zhenjiang 212013, China; zxl4390@126.com (X.Z.); wangbo@ujs.edu.cn (B.W.); m.shahzad.k@outlook.com (M.S.)

**Keywords:** soft sensor, fermentation process, monitoring and control, optimization

## Abstract

For effective monitoring and control of the fermentation process, an accurate real-time measurement of important variables is necessary. These variables are very hard to measure in real-time due to constraints such as the time-varying, nonlinearity, strong coupling, and complex mechanism of the fermentation process. Constructing soft sensors with outstanding performance and robustness has become a core issue in industrial procedures. In this paper, a comprehensive review of existing data pre-processing approaches, variable selection methods, data-driven (black-box) soft-sensing modeling methods and optimization techniques was carried out. The data-driven methods used for the soft-sensing modeling such as support vector machine, multiple least square support vector machine, neural network, deep learning, fuzzy logic, probabilistic latent variable models are reviewed in detail. The optimization techniques used for the estimation of model parameters such as particle swarm optimization algorithm, ant colony optimization, artificial bee colony, cuckoo search algorithm, and genetic algorithm, are also discussed. A comprehensive analysis of various soft-sensing models is presented in tabular form which highlights the important methods used in the field of fermentation. More than 70 research publications on soft-sensing modeling methods for the estimation of variables have been examined and listed for quick reference. This review paper may be regarded as a useful source as a reference point for researchers to explore the opportunities for further enhancement in the field of soft-sensing modeling.

## 1. Introduction

Biotechnology is based on the principle of using metabolic processes in microorganisms or cells to produce a desired product. For thousands of years, it has effectively been used to produce alcoholic beverages, dairy products or bread, and pharmaceuticals can be added to this list in the last century [1]. By using cells, e.g., microorganisms, as a plant to produce a desired product either by the cells own metabolism, or by introducing genetic information into the host cell and produce recombinant proteins. As in any type of process industry, there is a demand of knowledge, precise data and automatic feedback control to obtain a robust and consistent production process. The complex nature of chemical and biological processes makes this even more difficult and when it comes to pharmaceuticals, it places very high demands on quality. Automatic production process control has effectively been employed for several years in other related industries [2], for example computer-aided process control, which has established a new innovation that significantly expands the present scope of technical outcomes and it is expected to revolutionize many facts of agricultural, chemical manufacturing medical practices, and industrial. Biotechnology is currently in an incredible stage of development whose end is nowhere in sight.

According to the unique enzymological or bacteriolytic characteristics of the biological and chemical processes such as the marine alkaline protease MP fermentation, marine lysozyme fermentation, penicillin fermentation, L-lysine fermentation, it can be used in medicine, bioengineering, food preservation, and chemical industry [3,4,5]. However, these processes are usually complex nonlinear coupling systems with time-varying multiple variables. In order to realize the decoupling and optimizing control of the fermentation process, the key biological variables should be measured accurately in real-time. At present, the variables which can be automatically detected by appropiate instruments mainly center on some physical and chemical variables such as temperature, tank pressure, pH value, dissolved oxygen, stirring speed and so on. There are some essential key variables in the biological and chemical processes which have a critical influence on productivity such as cell concentration, substrate concentration, and product concentration that cannot be measured with practical online measuring instruments. The offline testing has a time-lag problem, and cannot satisfy the needs of real-time optimization control. Soft-sensing modeling is an important and effective way to solve the above problems [6,7]. A soft-sensing modeling introduction to the estimation of crucial variables for the process of fermentation is one of the hot research topics in the current academic and engineering field.

Soft-sensing technology has been a new and popular method in the field of monitoring in recent years. Its principle is to realize real-time estimation of quality variables that are difficult to measure directly through easily detectable auxiliary variables and some corresponding mathematical models. Therefore, soft-sensing technology has a very good effect on determining some difficult-to-measure variables in industrial processes like fermentation, and has great application potential. Currently, in the process industry or in the field of microbial fermentation, the commonly used soft-sensing methods are soft-sensing methods based on mechanism analysis, data-driven soft-sensing methods and soft-sensing methods based on hybrid modeling.

The purpose of this paper is to present a comprehensive review on the topic of soft-sensing modeling methods used for the process industry and shows their usefulness in improving robustness and increasing batch-to-batch reproducibility in process industries. Soft-sensing models and their applications are critically reviewed and classified in different categories such as support vector machine (SVM)-based soft-sensing models, neural network (NN)-based soft-sensing models, deep learning-based soft-sensing models, fuzzy logic (FL)-based soft-sensing models, genetic algorithm (GA)-based soft sensors, probabilistic latent variable models (PLVMs) and other useful methods. The already published reviews on soft-sensing modeling in the technical literature have not much focused on the most recent data-driven approaches as well as new optimization algorithms. In this review, more than 70 research publications on soft-sensing modeling methods have been examined, drawn in the form of figures separately, and listed for quick reference, so it may be regarded as a useful guide for researchers who are interested in the field of soft-sensing and wish to discover the opportunities offered by these methods for further enhancement in the field of soft-sensing modeling.

This review paper is divided into seven sections. Starting with an introduction in Section 1. Section 2 and Section 3 give a short introduction of soft sensor models and their development procedure. Reviews of soft-sensing modeling methods and optimization techniques are presented in Section 4 and Section 5, detailed descriptions/mathematical formulation of selected modeling methods are ignored here, and readers can easily find in the cited published papers and books which are listed in the references section. In Section 6, a comparative analysis of the soft-sensing modeling methods is described. Finally, future perspectives and some concluding remarks based on the review paper are covered in Section 7.

## 2. Soft Sensor

**Figure 1:**
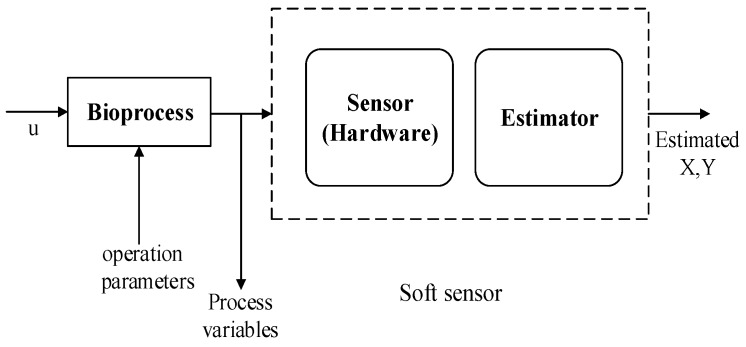
The concept of a soft sensor as presented in [10]. This figure describes the model of a soft sensor with one hardware sensor and an estimator. Input from a bioprocess is measured through the sensor (in reality this can be a combination of several hardware sensors) and new information is estimated.

The term soft sensor is a combination of two words: soft(ware) (i.e., computer programs) and sensor (a hardware counterpart). It is also known as a software sensor, virtual sensor, intelligent sensor, or inferential estimator. Software sensors are defined as the combination of hardware sensors and software models [6,7] that use well-measured variables to estimate process variables which are hard to measure directly because of technical limitations, measurement delays, and complicated environments. In the literature review, ‘difficult-to-measure’ variables are also referred to as target variables, dependent variables, or model output, while ‘easily-to-measure’ variables are referred to as process features, independent variables, or model inputs. Such soft sensors are generally employed in real-time process monitoring and control, modeling, fault diagnosis, etc. mainly in a continuous process. Nowadays, these soft-sensing methods are playing a key role in many industrial applications such as food processing, chemical plants, nuclear plants, oil refineries, the paper and pulp industry and surgical applications in various medical fields, etc., to estimate product quality parameters, and they have become a major developing trend in both academia and the industrial sector [8,9]. The soft sensor principle can be seen in Figure 1. As mentioned, this term is a combination of software and sensors. A soft sensor uses easy-to-measure process variables to predict unmeasurable bioprocess variables with software estimation algorithms.

## 3. Soft Sensor Development Methodology

In order to estimate the quality variables in real-time, one can use mathematical models, computation methods and previous knowledge to derive new process information. Today the concept is well established in engineering science and parts of the process industry. The soft sensor principle is based on hardware sensors monitoring a bioprocess in real-time and generating online data used by the soft sensor model to estimate process variables. The estimated process variables can then be used for monitoring purposes or be implemented in feedback control of the bioprocess. This section describes the typical steps of data-driven soft sensor model development. There are many steps in the development of soft sensor models in the literature [6,8], but in this review paper, soft sensor development has the following main steps: (I) data collection and pre-processing, (II) selection of variables, (III) soft-sensing model selection, training and validation and (IV) performance evaluation of the soft-sensing model. The presented soft sensor development procedure is quite general and can thus be applied to fermentation processes.

### 3.1. Data collection and Pre-Processing

During the initial stage of soft-sensor model development, an inspection of historical data is performed, which leads to better process understanding, diagnostics, and improvement. The goal of this phase is to recognize any obvious problems and attain an overview of the overall structure of data which may be managed at the initial stage of development. This paves the road to the subsequent use of collected plant data for model optimization, identification or other related black-box (e.g., data-driven) methods. The next goal of this phase is to ascertain the necessities for the model complexity. A well experience soft-sensing modeling developer can make a right decision at this stage whether we should use a simpl linear model, complex model or non-linear NN model for the soft sensor prediction model. Sometimes the decision of the soft sensor model developer may not be correct in the first stage, therefore the performance evaluation of the model should be compared with alternative soft sensor models at the later stage of development [6].

Apart from soft sensor model evaluation, the following is an important question to be addressed before we dive deeper into model development: how do you prepare the input data and output data or targets before feeding them into actual soft sensor model? The goal of this step is to turn the data in such a way that the actual model can process it more efficiently. This includes dealing with missing values, outlier’s detection and replacement, data normalization, and feature extraction [8,11].

The problem of missing values is very essential to understand in order to effectively manage data. This problem arises when no value is stored for one or more variables in an observation. There are distinct approaches to deal missing values. The first approach is the removal of samples containing null values. This approach is advised only when the number of missing values is small, otherwise will not give the expected results while predicting the output [12]. In the second approach, fill-in the missing values using some methods, for example, calculate either mean, median or mode of the feature and replace it with the missing values. These two approaches are quite common to deal with missing values. Some other useful approaches are also reported in the literature such as hot-deck imputation [13], maximum likelihood (ML), and expectation-maximization EM [14,15].

The observation values of the sensor are called outliers. Outlier values deviate from the typical or meaningful range of values. Outliers can be caused, for example, hardware failures, communication errors, incorrect readings, the process working conditions, and so on. This can affect the performance of the soft sensor model [6]. To alleviate the effects of outliers it is necessary first to identify them, and then to treat them. There are different outlier detection methods reviewed in the literature such as the 3σ outlier detection method [16], and the Hampfel identifier [16,17,18], which is a robust version of 3σ outlier method. The above methods are based on univariate outlier detection. In [8,19], the authors presented multivariate outlier detection methods. Some other multivariate methods employed in the soft sensor context is based on data projection such as Jolliffe parameters with PCA and PLS [8,20]. A comparison of different outlier detection approaches was provided in [21].

In NN and other data-driven soft sensor models we need to normalize the inputs; otherwise, the model will be ill-conditioned. There are different normalization methods reported in the literature such as min-max normalization, z-score normalization [8], and zero-mean normalization [22]. Data normalization means to have a value between 0 and 1, which is the simplest method of normalization [23,24].

The significance of pre-processing is important because of the data characteristics. Data pre-processing is the step that requires a large amount of manual work and expert knowledge about the underlying process. A more detailed discussion is provided in [25] regarding pre-processing approaches with real-world examples and their applications in the soft sensor context. A general overview of data pre-processing approaches is also presented in [26].

### 3.2. Selection of Variables

The very next question a soft sensor model developer faces is with regards to the selection of input variables that influence the model output. Generally, the number of input variables of soft sensor model should not be too many; otherwise the model structure will be for more complex, there will be a large probability of overfitting occurring, and it influences the model’s training speed and output. For NN modeling a reduction in the input variable leads to a simplified NN architecture and reduced training time [27]. There are many advantages in a reduced number of variables such as decreasing costs, reduction of model development time, and enabling the feasibility of an application. Many researchers have reported both supervised and unsupervised variable selection methods in the literature such as principal component analysis (PCA) [28,29], filter methods (correlation coefficient), wrapper methods, embedded methods [30], mean impact value (MIV) [31], and uniform incidence degree algorithms [24], to give just a few examples. However, in most of the cases, the selection of most relevant variables for many soft sensor applications is made by system experts. Nonetheless, the selection of variables is an effective procedure to increase the model performance, prevent overfitting, and also to avoid the curse of dimensionality phenomena. Special attention is given to the judgement of the output variables such as product concentration, cell concentration, and substrate concentration. The selected data is used for the training and evaluation of the soft sensor model.

### 3.3. Soft Sensor Model Selection, Training and Validation

A soft sensor ‘model’ can be a mathematical representation of a real-world process. Generally, a soft sensor model is organized into two distinct categories, such as mechanistic models (first principle or white-box) and data-driven (empirical or black-box) models. The mechanistic models belong to the first category of soft sensors which are based on the first principle approach. Usually, mechanistic soft sensor models focus on the description of the optimal steady states. This type of soft sensor is mainly based on deriving equations that can describe the physical and chemical background of the process. For example, the Kalman filters and extended Kalman filter techniques belong to this category. The major disadvantages of mechanistic models is that they are considered to be rather time-consuming, as most of the processes are very complex and they are unable to express the actual process conditions [9]. As a result, the data-driven soft-sensing modeling algorithms are becoming progressively popular in the process industry [6] because data-driven soft sensor models are based on empirical observations and thus describe the real conditions of the process. Moreover, it requires few knowledge about the system to be modeled, describes the input and output relation more accurately, and producing reliable real-time estimation of unmeasurable process variables in the process industry. For example, SVM, NN, FL methods belong to the second category of soft sensor models.

For soft-sensing modeling, the selection of a model is always an important matter that needs to be paid much attention. Model selection is the critical process of selecting one final model from among a collection of candidate models for the training dataset. As the model is the heart of a soft sensor, the selection of the optimum kind is important for the soft sensor’s performance. The choice of models is also often subject to the personal preference and past experience of the developers that can be of a drawback for developing an optimum final soft sensor. This can be seen in the subject of published applications for soft sensors, where several researchers concentrate strongly on a model type in their field of expertise. Just to give a few examples, if the process variables are non-Gaussianly distributed or they have a non-linear relationship with each other, a non-linear probabilistic latent variable modeling method needs to be utilized; If the process variables are Gaussianly distributed or they have a linear correlation with each other, then a linear Gaussian probabilistic/PCA should be employed [32]. Deep neural network models demonstrate great performance in complicated highly-non-linear processes, comprising richer information in deep layers of network and large training datasets [33]. With the advantage of a small amount of data, SVM and LS-SVM enjoy high efficiency and robustness and thus have been commonly used [34]. Another possible way for this task is to start with a simple model like linear regression and gradually increase the complexity of the model until we consider that we have a good model [6,35].

A large number of samples and the complex nature of the soft-sensing modeling methods need very long training times. As such, it is typical to use a simple separation of sample data into training and validation or training and test samples. By using the training samples, we train our selected soft-sensing model (used to fit the model) and evaluate the model on the validation/test data. It is essential to evaluate the soft sensor model performance on independent data while performing this task. There are different approaches [36,37] to estimate the performance of soft sensor model on unseen data such as automatically splitting a training dataset into train and validation datasets, manually and explicitly, and evaluating the performance by using k-fold cross-validation.

### 3.4. Performance Evaluation of Soft Sensor Model

After selecting the optimal soft sensor model structure and training the model, a trained soft sensor model has to be evaluated on an independent/test dataset once again. The test data provides the gold standard used to evaluate the soft sensor model. However, how to evaluate the performance of the data-based model is still an open question because performance evaluation is highly related to the selection of the soft sensor type. There are different criteria’s available for performance evaluation of each monitoring model. In the case of numerical performance evaluation, mean square error (MSE) or root mean square error (RMSE) loss function commonly used for such type of soft sensor models. Recently, in [38] the authors proposed a novel fuzzy decision fusion system based on an analytic hierarchy process (AHP) for online process monitoring. Seven different tools were suggested for the evaluation of the model performance and tested on six different data-based process modeling methods.

The methodology presented in this review paper is the one most commonly used but is not the only possible way for developing a soft sensor model. For example, the presented methodology of a soft sensor model in [8] and [6] is detailed but consistent with our discussed methodology. In [39], in addition to a general five-step soft sensor development methodology consisting of: (i) the collection of historical data and pre-processing, (ii) variables selection, (iii) model selection and training, (iv) validation, and (v) model maintenance, is more detailed but consistent with the methodology discussed here. There is a significant difference between the discussed methodology and in other presented works in that we have explained all the development procedures with the help of a real-time example of the microbial fermentation process.

### 3.5. Use Case Implementation of Soft Sensor

In this review paper, a penicillin fermentation process is taken as a research example for a better understanding of the development procedure of a soft sensor model. An experimental study was conducted on the penicillin fermentation process in the biological fermentation tank of the “National Key Discipline” laboratory of Jiangsu University. As we know, the penicillin fermentation process is a time-varying and complex nonlinear biochemical process. There are many input variables (auxiliary variables) available in the fermentation process, such as dissolved oxygen, CO_2_ concentration, temperature, reactor volume, pH value, stirring speed, substrate given rate, and so on. If all of them listed as input variables, the proposed model would be more complicated and affect the training speed. In order to determine the impact of input variables on soft-sensing model output, the concept of incidence degree is employed to assess the incidence degree among input and output variables.

Every one minute the field test data is sampled: glucose flow *f*_g_ (*u*_1_), corn pulp flow rate *f_cs_*(*u*_2_), flow rate of potassium dihydrogen phosphate *f_p_*(*u*_3_), calcium carbonate flow rate *f*_c_(*u*_4_), flow rate of gluten powder *f_r_*(*x*_5_), dissolved oxygen concentration *C_L_*(*x*_4_), carbon dioxide concentration *Cco_2_*(*x*_5_), [H^+^]concentrations [H^+^](*x*_6_), and fermentation broth volume *V*(*x*_7_). Every four hours, the offline biological variables are obtained by sampling and testing: cell concentration *X*(*x*_1_), substrate concentration *S*(*x*_2_), and product concentration *P*(*x*_3_). The cell concentration, substrate concentration, and product concentration are selected as the target variables. The growth of biomass cells depends on several environment factors involving all the types of input control variables. Substrate concentration was tested with a glucose analyzer and a UV altimeter is used to measure product concentration. A total of 10 batches of fermentation process data were collected over a 200 hour span (between each batch). In order to improve the measurement accuracy, the sample data should be normalized within the range of [0,1].

**Figure 2:**
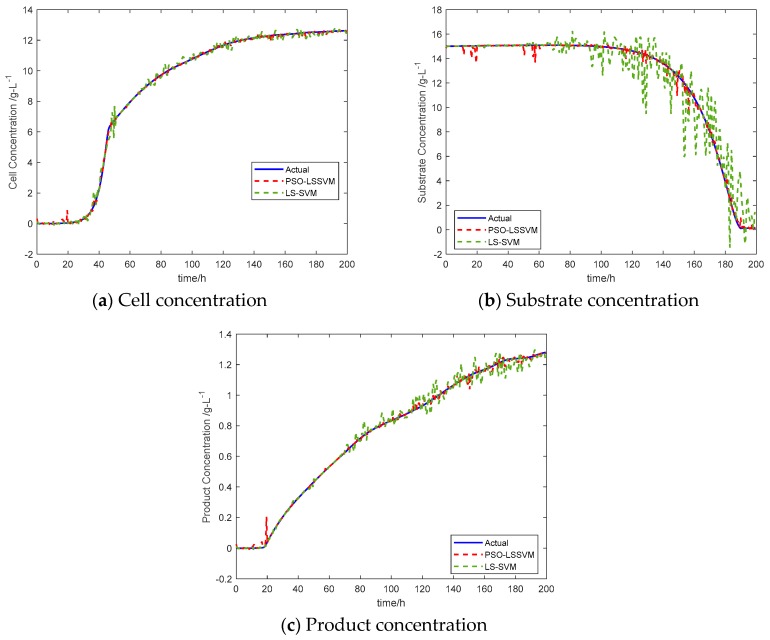
Comparison of soft-sensing results.

**Figure 3:**
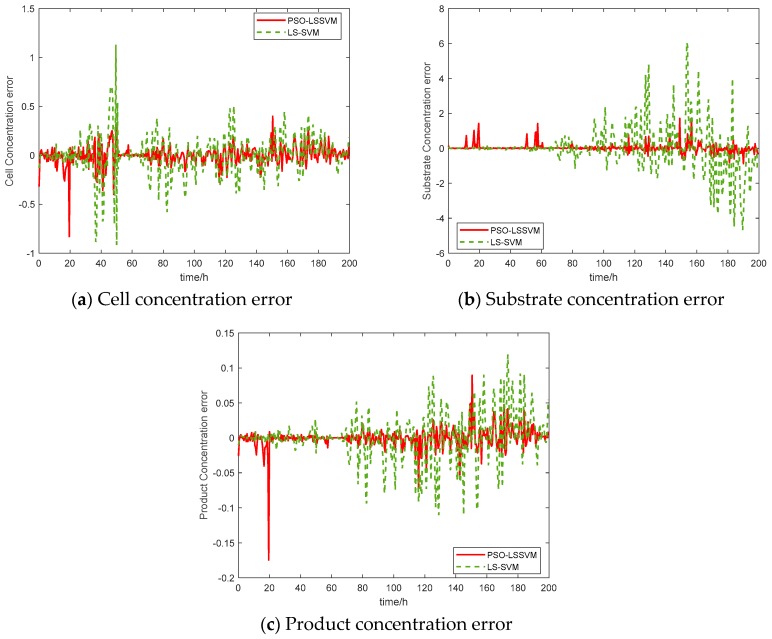
Error curve comparison diagrams.

In recent years, many data-based techniques have been introduced for real-time estimation or process monitoring and fault detection, where every technique performs well under its own assumption. In other words, a technique that performs well in a certain process condition may not provide a reasonable performance under several other process conditions, because of different data features [38]. This review paper proposes a soft-sensing model based on Least Square Support Vector Machine (LS-SVM). The proposed model is successfully applied to the estimation of cell concentration, substrate concentration, and product concentration in a penicillin fermentation process. LS-SVM is a machine learning method based on statistical learning theory. It has excellent learning ability and prediction ability with small sample data, low difficulty in training and has been widely used to predict the quality variables in the fermentation process, steel, chemical and other industries [40,41]. Practice demonstrates that the values of the kernel parameters and penalty parameters of LS-SVM play a significant role in the generalization ability and accuracy of the model, and improper selection may make the LS-SVM prediction model prone to over-fitting and poor generalization ability. To solve this problem, many researchers have developed several optimization algorithms for LS-SVM model parameter selection. In this work, the parameters (e.g., regularization parameter C and gamma) of the LS-SVM model are optimized by using an evolutionary algorithm, namely particle swarm optimization (PSO). The basic idea of the PSO algorithm is to discover the global best solution through provided information and sharing among individuals in a group. A total of 10 batches (2,000 samples) of fermentation data were used in this example, among which the first six batches (1200 samples) of the experimental data were used to train the model for minimum error. The 7th and 8th batch (400 samples) of the experimental data is used for k-fold cross-validation and the data of the last two batches was used to test the final soft sensor model. The optimization algorithm has been iterated many times to attain a global optimal point. The actual and predicted results of the soft-sensing model based on PSO-LS-SVM and LS-SVM are shown in Figure 2, these results are compared with the LS-SVM model to verify the effectiveness of the soft-sensing model. Figure 3 presents the error curve between the PSO-LS-SVM soft-sensing value and the LS-SVM value. The simulation results demonstrate that the prediction results of PSO-LS-SVM soft-sensing model are closer to the real values.

Once the soft sensor model is ready for prime time, we test it one final time on the test dataset. In this example, the proposed model is used to train the data, which verified the fitting degree and prediction accuracy with the test dataset, and we select the mean absolute error (MAE) and root mean square error (RMSE) as the evaluation criteria for model performance. Table 1 displays the prediction MAE and RMSE results of the PSO-LS-SVM soft-sensing model and the LS-SVM soft-sensing model on the test dataset. It can be seen that the error difference of the PSO-LS-SVM model is less than that of the the LS-SVM model. This is because of the optimization algorithm used, as the PSO-LS-SVM modeling method has excellent learning ability and prediction ability with small sample data and is suitable for the penicillin fermentation process. A more detailed discussion on optimization techniques will be described in Section 5.

## 4. Review of Modern Soft-Sensing Models and Optimization Techniques

**Figure 4:**
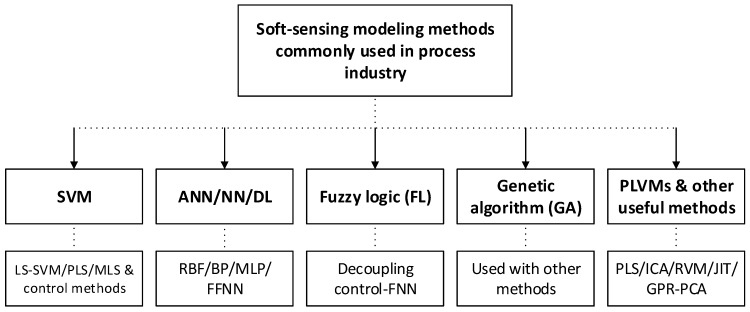
Soft-sensing models used in process industry (abbreviations used are listed in Table A1).

This part presents an in-depth review of artificial intelligence-based soft-sensing models (e.g., data-driven models) and research on model optimization for different kinds of industrial processes. AI or machine intelligence merges a wide variety of innovative technologies to give machines decision making, problem-solving, learning, perception, and reasoning ability [42]. This is achieved using technologies such as those we mention in the section below. The tools and methods reviewed here are ones that have proved to be useful with a sensor system. Currently, with profound studies into the theory of soft sensor control and the ongoing advancement of engineering technology, some modern methods have appeared and developed rapidly to solve the problems which are hard to measure for soft sensor models, such as FL [43], partial least squares (PLS) [44], SVM [45], support vector regression (SVR), radial basis function (RBF), NN [23], GA [46], and PLVMs [32]. Some important soft-sensing modeling methods, shown in Figure 4 are used for the prediction of quality variables. These novel methods have been widely used across many fields of the process industry such as the chemical, pulp and paper and steel industry. The most common applications of soft sensors are the prediction of values, process monitoring and control, and fault detection. A short summary of each will be discussed as a soft-sensing model with different fermentation objects in the following part.

### 4.1. Support Vector Machine-Based Soft-Sensing Models

**Figure 5:**
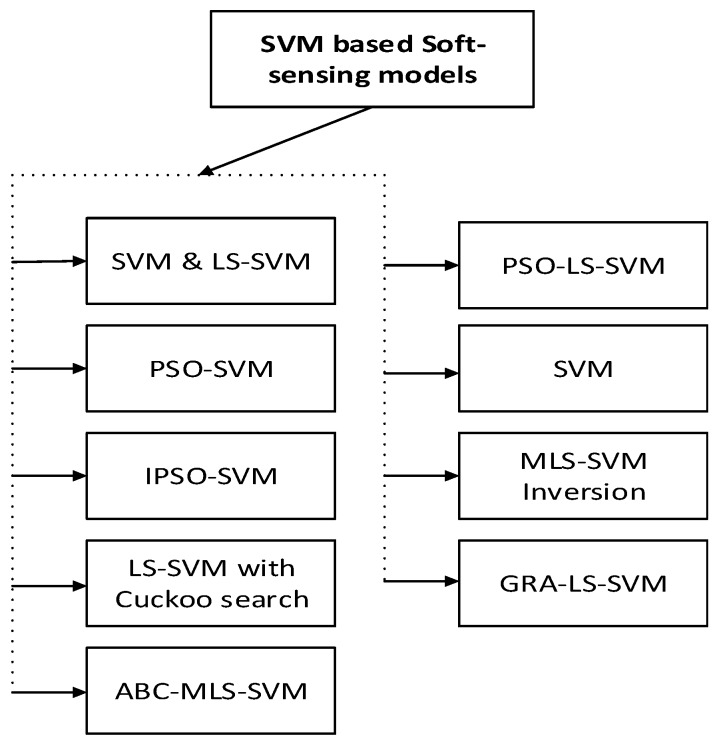
SVM based soft-sensing models (abbreviations are listed in Table A1).

The most commonly used driving modeling methods mainly include modeling methods based on SVM. This is a supervised machine learning algorithm that is used for regression problems, classification, and time series prediction, as originally introduced by Vapnik [47]. The SVM algorithm is based on the concept of structural risk minimization. It is a kernel-based method that enables linear, polynomial, and radial basis functionality (RBF) to be used and others that fulfill Mercer’s condition. SVM has the characteristics of being able to fully approximate arbitrarily complex nonlinear systems, and have the ability to self-learn and adapt to the dynamic characteristics of uncertain systems. SVM has excellent learning ability and prediction ability of small sample data and is suitable for the fermentation process with fewer sample data. In SVM, giving a training dataset *d* = (*x_i_*, *y_i_*), *i* = 1, …, *n*, where n indicates the number of samples. *X_i_* ∈ *R^d^* are multi-dimensional inputs, *y_i_* ∈ *R* is the continuous output data. We seek to locate a continuous mapping function *f* : *R^N^* → *R* that best predicts the set of training points with the function *y* = *f*(*x*). SVM based soft-sensing models are shown in Figure 5.

In [45], the authors presented a soft-sensing model based on the SVM and LS-SVM for microbiological fermentation processes. LS-SVM was proposed by [48] using the idea of SVM, which is used to solve the problem of model decomposition and function estimation. The LS-SVM soft-sensing model is selected due to an evidence for better generalization properties when compared to an NN. In [49], authors apply the presented SVM black-box model to the estimation of quality variables of antibiotic fermentation process. They used the Gaussian and polynomial function as a kernel function with SVM. Kernel function takes data as input and transfers it into the required output. SVM soft-sensing model was compared with the back-propagation NN model. SVM proves effective to tackle problems with small datasets, while for large training samples the computation cost is unaffordable. In [50], the authors proposed the generalized predictive control (GPC) method based on LS-SVM. The particle swarm optimization algorithm was applied for the optimization of the regularization parameter ‘C’ and kernel parameter ‘g.’ The performance was tested in terms of a bacteria concentration prediction in a marine lysozyme fermentation process. The PSO-LS-SVM soft-sensing model was compared with the LS-SVM model. In [51] authors developed and published a soft-sensing model based on PSO-SVM. The performance of developed model is demonstrated by applying it to biomass concentration in a lipid fermentation process. PSO-SVM soft-sensing model was compared with the SVM model.

In a recent publication [52] a robust decoupling control method based on Multiple LS-SVM inversion system has been proposed to the prediction of the quality variables (e.g., biomass concentration, substrate concentration, enzyme concentration) in a marine alkaline protease MP fermentation process. An intelligent optimization algorithm, namely artificial bee colony (ABC) is used to optimize the MLS-SVM model parameters. The ABC algorithm is in more detail described in Section 5. The proposed ABC-MLS-SVM based soft-sensing model has been compared with PID control. Another very popular decoupling control method based on the LS-SVM inversion system was proposed and published in [53]. The developed method is used for the modeling of an L-lysine fermentation process. The authors were satisfied with the performance of the presented soft-sensing model.

In [54] researchers proposed a gray relational analysis LS-SVM soft-sensing model for glutamate concentration. The GRA-LS-SVM soft-sensing model is compared with the RBF-NN model. The authors of the above paper have presented another soft-sensing model based on Multi-Phase SVR for the prediction of quality products in glutamate fermentation [55]. It is obviously demonstrated that the performance of the presented soft-sensing model is superior to other models. The authors also define the theoretical description of the performance advantages. In [56] authors introduced the improved version of the PSO algorithm (IPSO) to the selection of optimal parameters for the mixed kernel function of the SVM model. In [57], studied the development of a soft-sensing model based on LS-SVM to estimate the unmeasurable variables in industrial procedures. LS-SVM soft sensing model is compared with the RBF-NN model.

In [58], a soft-sensing modeling method based on multiple output variable least squares support vector machine (MLS-SVM) is published. This soft sensor is based on a combination of the inverse system and SVM theory. The authors apply the presented model to the estimation of key variables (e.g., product concentration, substrate concentration, biomass concentration) of L-lysine fermentation process. The MLS-SVM inversion soft-sensing model was compared with the LS-SVM model. The soft-sensing modeling method based on an accurate incremental online v-SVR learning algorithm suggested in [59] for the concentration of biomass during the fermentation process. An important data-driven soft-sensing model based on iteratively weighted LS-SVR by using a cuckoo search (CS) optimization algorithm presented in [60]. Comparisons of different SVM based soft-sensing modeling methods are provided in Table 2.

### 4.2. Neural Network-Based Soft-Sensing Models

**Figure 6:**
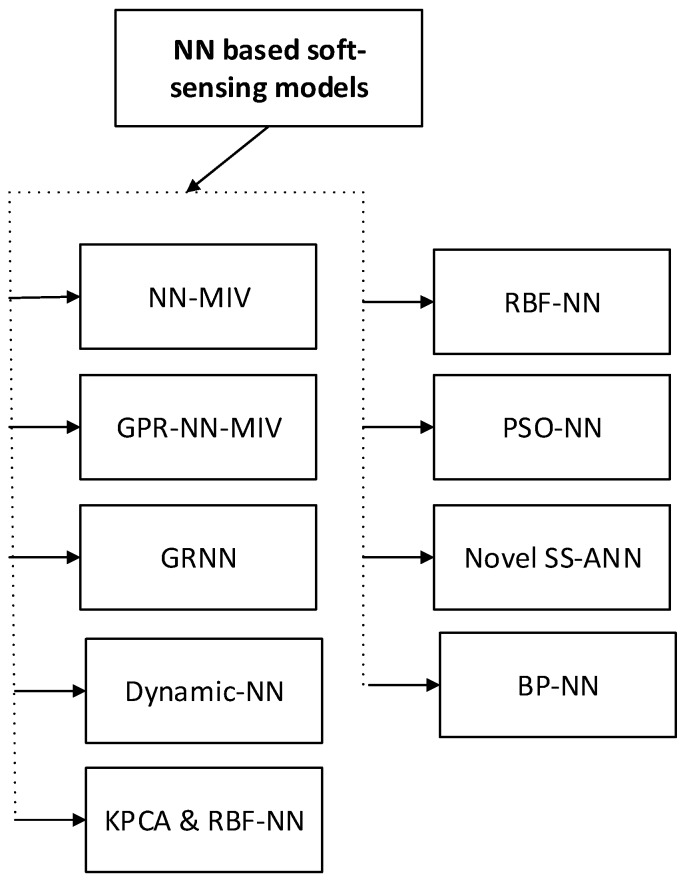
NN-based soft-sensing models (abbreviations are listed in Table A1).

A neural network consists of input and output layers, as well as (in most cases) a hidden layer consisting of units that transform the input into something that the output layer can use. NN has been implemented effectively to a wide spectrum of areas, including data mining, geology, finance and insurance, forecasting, physics, engineering, biology,medicine, and other industrial applications. They are excellent tools for regression, classification, data clustering, optimization and finding patterns that are far too difficult or numerous for a human programmer to extract and teach the machine to identify. The soft-sensing modeling methods based on NN has shown in Figure 6.

In [31], the NN-MIV soft-sensing model was presented for the estimation of the key variables (e.g., marine enzyme activity) in a marine enzyme fermentation processf. The NN-MIV soft-sensing model was compared with the NN model. Another GPR-NNMIV based soft-sensing model was presented in [61]. In this work NN-MIV variable selection method is employed to get the most suitable input variables with the highest contribution rate. Finally, the presented model based on NN-MIV with gauss process regression (GPR) is used to the prediction of the marine enzyme fermentation process. The GPR-NNMIV model performance is compared with a single GPR model, the authors conclude that the presented method is capable of attaining a satisfactory prediction performance. In [23], the authors proposed the RBF-NN soft-sensing model to the estimation of the key variables (e.g., cell concentration, enzyme activity, matrix concentration) of marine alkaline protease MP fermentation process. A Gaussian function used as the basis function and method of formula and coincidence degree algorithm used for the selection of input variables. In [62], studied the development procedure of PSO-NN soft-sensing model. In [63], the authors proposed the robust NN based soft-sensing modeling method for the biomass concentration in the process of fermentation. K-nearest neighbors (KNN) algorithm used for the calculation of the anomaly degree of each modeling data set and the weight of each modeling data sets are decided by the computed degrees of the anomaly. In [64], presented a BPNN soft-sensing model for the lysine fermentation process.

Another soft-sensing modeling method based on ANN was published in [65]. An ANN offers the opportunity to deploy neurons which denote the process knowledge. The authors apply the presented ANN soft-sensing model to the estimation of crucial variables (e.g., mycelia concentration, sugar concentration, and chemical potency) of the erythromycin fermentation process. In [66], established the generalized regression NN soft-sensing model to estimate the key biological variables (e.g., substrate concentration, biomass concentration, enzyme activity) which are hard to measure in the marine protease fermentation process. The GRNN soft-sensing model has been compared with RBFNN. The researchers in [67] developed the feature extraction approach based on kernel principal component analysis (KPCA) for nonlinear data, RBFNN method used in the modeling of biomass estimation in the process of fermentation. In [68], the authors proposed the dynamic NN soft-sensing model to control the enzyme activity during the fermentation process. The comparison of different NN based soft-sensing modeling methods is provided in Table 3.

### 4.3. Deep Learning Based Soft-Sensing Models

Various statistical and machine learning methods have effectively been utilized for data-driven soft-sensing modelings, such as SVM, ANN, PCR, and PLS etc. SVM proves effective to tackle problems with small datasets, while for large training samples the computation cost is unaffordable. ANN is a machine learning technique extensively applied in pattern recognition, which has a non-linear structure and can approximate a nonlinear continuous function by arbitrary precision, and thus is suitable for nonlinear industrial process, but the problem is that the traditional gradient descent method is slow to converge, and the noise in industrial data often causes local optima for ANN. In recent years, deep learning based models are becoming more and more prominent in many fields such as computer vision, speech recognition, image classification, bioinformatics, natural language processing (NLP) and several other domains [69,70,71]. Deep learning, a subset of machine learning, is capable of learning deep, hierarchical artificial neural networks efficiently. Compared to the traditional methods like SVM and ANN, deep learning has more depth of layers, advance convergence method and stronger ability to approximate [33].

In [72], the data-driven soft-sensing modeling method based on deep learning has been introduced for the estimation of critical variables of streptokinase and penicillin fermentation processes. In this work, a semi-supervised and supervised strategy is employed by using the unlabeled and labelled dataset. The performance of the proposed method is compared with SVR model. The authors are satisfied with the performance of deep architecture because it performs better for the large training dataset. The deep probabilistic latent variable regression model based on variational auto-encoder (VAE) is developed in [73] for soft-sensing application in the process industry. The VAE is a deep generative model which can deal with complex nonlinear relationships among different variables. In [74], the authors proposed the deep neural network (DNN) structure based on long short-term memory (LSTM) as a soft-sensing model to deal with strong nonlinearity and dynamics of the industrial process. LSTM is a recurrent neural network (RNN) architecture which contains memory and forgetting structures. A semi-supervised deep learning model based on the hierarchical extreme learning machine (HELM) for the estimation of critical quality variables is presented in [75]. The HELM contains the supervised and unsupervised feature extraction components and it can be used in regression problems and data classification. In [33], the deep learning method is employed to build a soft-sensing model and applied to an industrial case to estimate the process variables. In [76], the authors presented a soft-sensing modeling method based on a deep learning, which integrates denoising auto-encoders (DAE) with SVR. In another paper [9], authors utilized the deep learning method with DAE and improved version of the gradient descent algorithm is employed to update the model parameters. In [77], the authors suggested the soft-sensing model based on stacking auto-encoders (SAE) in the way of deep learning for the prediction of crucial process variables. Deep learning proves to be a promising method for soft-sensing modelling in highly data-driven complex bioprocesses.

With the recent development of advanced technologies, like the internet of things (IoT), smart devices, wireless sensors, wireless communications and data acquisition systems, the large amount of data collected from the process and stored in the industrial database. The era of big process data has arrived [78,79]. Therefore, for data modeling and monitoring large-scale processes with big data form multiple operating conditions, novel methods have been developed by many researchers. In [80] authors proposed the distributed parallel process modeling method based on a MapReduce structure for the quality prediction of large-scale datasets. A MapReduce is a programming framework which can be used for the file storage system and data processing [81,82]. In [83] authors presented a distributed parallel probabilistic modeling method based on scalable parameter server (PS) framework for big data quality prediction. An efficient scalable PS is a distributed computing architecture which is based on SGD optimization algorithm [84,85] and it can be used to handle industrial big data. A novel variational inference semi-supervised Gaussian mixture model (VI-SSGMM) have been employed in [86] for big data quality prediction. The presented method can efficiently take benefit of the extra information contained in the unlabeled data to learn the data patterns, which would improve the prediction accuracy of the model. In many soft-sensing applications, labeled data are usually limited due to technical or economic reasons, which adds obstacles to model training. To overcome the above problems, some semi-supervised machine learning methods have been developed by researchers and examples are addressed in this review paper.

### 4.4. Fuzzy Logic Based Soft-Sensing Models

**Figure 7:**
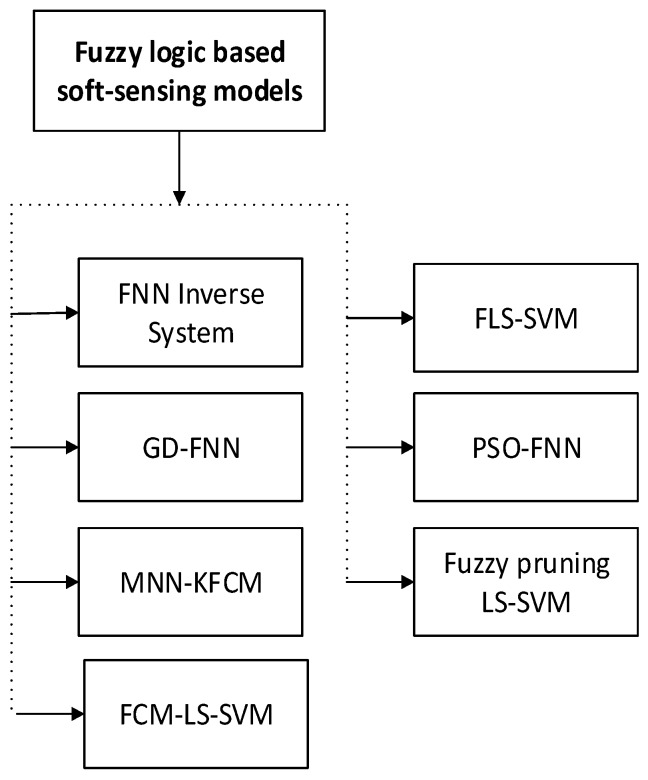
FL-based soft-sensing models (abbreviations are listed in Table A1).

FL is a technique of reasoning that resembles human intellect. The method of FL emulates the way of decision making in humans that includes all intermediate chances between digital values 1 and 0. The idea of FL was first invented by [87]. New computing techniques based on FL can be applied in the advancement of intelligent approaches for decision making, identification, pattern recognition, optimization, and control [88]. The soft-sensing modeling methods based on FL has shown in Figure 7.

A decoupling control method based on a fuzzy neural network (FNN) inverse system was proposed and published in [43]. The proposed soft-sensing model merges the inverse system theory with the intelligent control method. The performance was tested in terms of mycelia concentration prediction, substrate concentration, and relative enzyme activity in a marine biological enzyme fermentation process. The proposed system is compared with the NN model, PID controller, and some other control methods.

The researchers in [24] developed the generalized FNN soft-sensing model to the estimation of key biological and chemical variables (e.g., product concentration, substrate concentration, and biomass concentration) of the penicillin fermentation process. Uniform incidence degree algorithm used to identify the input variables (e.g., dissolved oxygen, carbon dioxide, the flow rate of glucose).

Another method presented to soft-sensing modeling is the Generalized Dynamic fuzzy neural network for microbial fermentation processes [89]. In [90], the authors proposed the multi-model NN soft-sensing model based on modified kernel fuzzy clustering for the erythromycin fermentation process. Feature selection done by PCA and fuzzy c-means clustering (FCM) algorithm based on PSO is applied to group the principal data into overlapping clusters, and NN is used to build sub-models based on the clusters. In [91], a fuzzy LS-SVM (FLS-SVM) soft-sensing model for the lysine fermentation process was presented. In [92], the authors proposed the soft-sensing model based on PSO-FNN for the lysine fermentation process. In [93], the fuzzy pruning LS-SVM soft-sensing model for the microbial fermentation process was presented. Another very common and successful family of approaches employed to soft-sensing [94] are the FCM algorithm and LS-SVM theory. In this work, FCM is utilized for separating whole training samples into many clusters and every subset is trained by LS-SVM model. The authors apply the presented model to predict the biological variables (e.g., mycelia concentration and relative enzyme activity) in the marine alkaline protease fermentation process. The comparison of different FL-based soft-sensing modeling methods is provided in Table 4.

### 4.5. Genetic Algorithm-Based Soft Sensors

GA is a technique for solving optimization problems that are based on the mechanics of natural selection and natural genetics. GA is a computing search algorithm used to discover accurate or approximate results to optimization and search problems. At each phase, GA chooses an individual to be a parent at random from the present population and use them for the next generation to produce children. Over consecutive generations, the population evolves towards an ideal solution. The GA is considered to be an excellent intelligent paradigm for optimization using a multipoint, probabilistic, random and guided search mechanism [95].

In [96], the authors described a SVR soft-sensing model based on GA and Akaike information criterion (AIC) for the erythromycin fermentation process. An estimation of the fermentation process variables (e.g., biomass concentration) by using the GA-SVM soft-sensing model was presented in [97]. In [98], the authors proposed the GA-BPNN soft-sensing model for the germ concentration in the process of fermentation. In another paper, a soft-sensing modeling method based on GA-BPNN was presented [99]. The hybrid model based on GA and an ant colony optimization (ACO) is introduced in [100] for the fed-batch fermentation process. The hybrid GA-ACO model was compared with the conventional GA and stand-alone ACO model. The comparison of different GA based soft-sensing modeling methods is provided in Table 5.

### 4.6. Probabilistic Latent Variable Modeling Methods

Due to the high-dimensional nature of the process industry data, dimensionality reduction is always needed, otherwise, data analysis could be quite difficult. As a solution, numerous data-driven regression modeling methods based on latent variables such as partial least square (PLS) and principle component regression (PCR) have been extensively applied for soft-sensing applications [101,102,103,104]. By projecting the process data into a lower-dimensional space from higher-dimensional space, latent variable modeling methods would be able to extract the crucial information from the industrial data. Nowadays, latent variable models have been rebuilt through probabilistic framework by many researchers, for example, probabilistic PCA and probabilistic PCR [105,106,107]. The probabilistic data model has several additional benefits on traditional methods. First, the expectation-maximization (EM) algorithm can be used for the estimation of probabilistic models parameters. Second, the probabilistic model can handle the problem of missing values in a practical dataset. Third, a single probabilistic modeling structure can be straightforwardly extended to the mixture form, which can be used for more complicated cases. Furthermore, probabilistic modeling can formulate Bayesian regularization methods which can be used to automatically determine the dimensionality of the latent variable model. Latent variable modeling methods have been employed for discriminant analysis, clustering, process data monitoring, regression modeling, and classification etc. There are different probabilistic methods have been reviewed in the literature such as probabilistic PCA, probabilistic independent component analysis (ICA), probabilistic PLS, and factor analysis. A more detail discussion and research status of different kinds of PLVMs is provided in [32].

The authors in [108] proposed the dynamic PLVM for regression modeling and soft-sensing application in industrial processes. The EM algorithm has been utilized for parameter estimation of dynamic PLVM. In [80] the authors presented the semi-supervised probabilistic principal component regression (SSPPCR) model for big data quality prediction in the process industry. The semi-supervised mixture of latent factor analysis (SSMLFA) model based on efficient EM learning algorithm is presented in [109] for the estimation of key quality variables. In [110], a soft-sensing modeling method based on semi-supervised probabilistic mixture of extreme learning machine (SSPMELM) was developed for monitoring and control of crucial variables. The adaptive soft-sensing model based on streaming parallel variational Bayesian supervised factor analysis (SP-VBSFA) model is proposed in [111] for quality prediction with big process data.

### 4.7. Other Useful Methods

**Figure 8:**
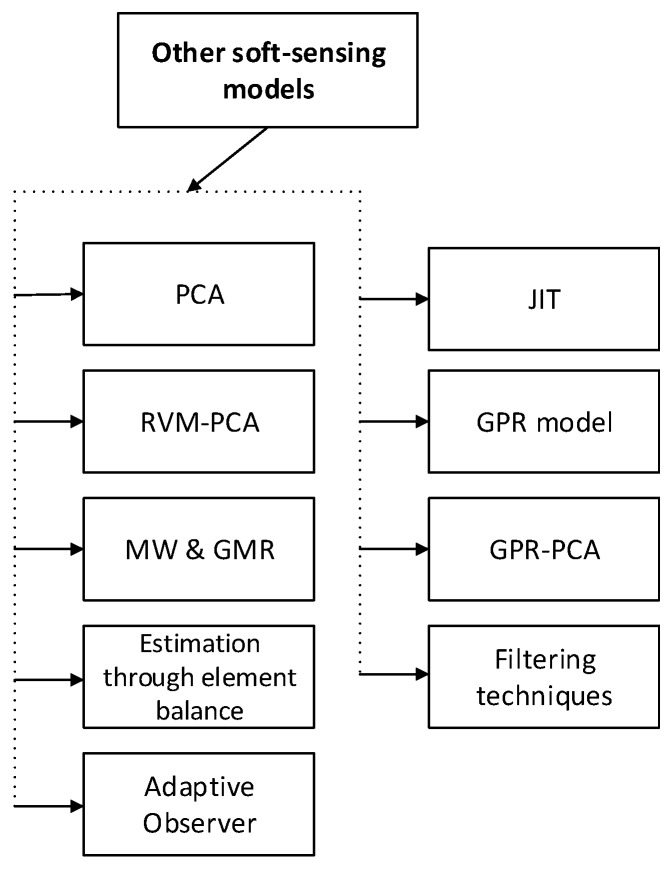
Some other useful methods (abbreviations are listed in Table A1).

In industrial process control, soft sensors have been commonly used to enhance product quality and ensure manufacturing safety. Some other useful soft-sensing modeling methods shown in Figure 8.

The following methods are also used for soft-sensing modeling:

- Filtering techniques, estimation through element balance and adaptive observer [112].
- Relevance vector machine (RVM) based on the PCA algorithm for fermentation [113].
- Gaussian mixture regression (GMR)-based soft-sensing modeling method presented in [114].
- Soft-sensing modeling methods based on multi-model adaptive by using local learning and online SVR for non-linear time-variant batch processes [115].
- Just-in-time (JIT) modeling with a combination of input and output similarity criteria for the soft-sensing modeling in fermentation processes [116].
- Dual learning-based online ensemble regression approach for adaptive soft-sensing modeling of nonlinear time-varying processes [117].
- A soft-sensing model based on GPR for the erythromycin fermentation process is presented in [118].
- A soft-sensing modeling method based on multi-model strategy by using GPR and PCA is presented in [119].
- A soft-sensing modeling method for product concentration monitoring in a fed-batch fermentation process based on dynamic principal component regression PCR proposed in [120].

## 5. Optimization Techniques

In order to ensure a better prediction accuracy of the model, the data-driven soft-sensing modeling methods need to use some optimization algorithms to optimize the model parameters. In this review paper, the optimization techniques used for the estimation of optimal parameters such as PSO, ABC, ACO, CS algorithm, and GA, are also discussed. PSO is an evolutionary algorithm, which was introduced by [121,122] based on swarm behavior such as birds flocking and fish schooling in nature. The idea of the PSO algorithm is to discover the global best solution through provided information and sharing among individuals in a group. In Zhu and Zhu [50], authors utilized the PSO algorithm with GPC-LS-SVM soft-sensing model. In another research paper, the improved version of PSO was utilized by [56], just given a few examples.

The ABC algorithm belongs to the evolutionary family, and it can be applied for solving numerical problems, optimization in dynamic and uncertain environments. The ABC optimization algorithm was developed by [123], in which the author has simulated the behavior of honey bees. ABC has three types of honey bees: workers, onlookers and scouts. The onlooker bees reside at the dancing area to decide about food source selection after evaluating the received information, and worker bees are those who provide information to onlookers and moving towards the food source (solution) visited by themselves earlier. The random search is performed by scout bees. Only a single worker bee is assigned to a single food source, and after collecting the detailed information in the first half of the algorithm, it shares information with onlooker bees that perform their job in the second half. The worker bee will become a scout after its food source is completely exhausted. The authors of this paper [52] have been utilized the ABC optimization algorithm with the Multiple LS-SVM inversion soft-sensing model.

The ACO is a population-based optimization technique. It was inspired by the ethological studies on the foraging behavior of ants. The ACO technique is implemented by instantiating a team of software agents, which simulate the ant’s behavior, walking around the graph representing the problem to solve [124]. The authors in [100] utilized the ACO algorithm to the parameter identification problem in the fed-batch fermentation process.

The CS optimization algorithm belongs to the evolutionary family and it was proposed by [125]. The CS algorithm was motivated by the breeding habit of cuckooc that chose a host nest to lay their eggc. The host may realize that it is an alien egg, and in that case, it may discard the strange egg or abandon it to construct a new nest. It uses the Lévy flight concept to update the nest position, which follows a random walk that is based on a truncated probability distribution step size [126]. In [60], the authors presented a data-driven soft-sensing model based on iteratively weighted LS-SVR with a CS optimization algorithm.

GA is a technique for solving optimization problems that are based on the mechanics of natural selection and natural genetics. GA is used to discover accurate or approximate results to optimization and search problems. At each phase, GA chooses an individual to be a parent at random from the present population and use them for the next generation to produce children. Over consecutive generations, the population evolves towards an ideal solution. GA based soft-sensing models have been discussed above.

## 6. Comparative Analysis

A large number of soft-sensing modeling methods have been discussed in this paper. The literature review demonstrates that the various optimization and data-driven methods have been combined together to estimate relevant process variables. The comparison study of some of the main soft-sensing modeling methods is given in Table 2, Table 3, Table 4 and Table 5, respectively. According to the developed review, it can be concluded that commonly used methods for the process of fermentation are, NN-, deep learning-, FL-, PLVMs-, SVM-based regression and hybrid methods. The comparative study of these methods will help in selecting the particular method for the fermentation process or specific application.

## 7. Conclusions and Future Perspective

Generally, soft-sensing modeling methods are becoming more and more popular in a broad range of industrial applications such as process monitoring, process control, fault diagnostics, and so forth. Such methods are very valuable to apply when physical or hardware sensors cannot be employed or when direct measurements are not accessible. In this paper, a comprehensive review of existing data pre-processing approaches, variable selection methods, data-driven (black-box) soft-sensing modeling methods and optimization techniques was carried out. The data-driven methods used for the soft-sensing modeling such as support vector machine, multiple least square support vector machine, neural network, deep learning, fuzzy logic, probabilistic latent variable models are reviewed in detail. The optimization techniques used for the estimation of model parameters such as particle swarm optimization algorithm, ant colony optimization, artificial bee colony, cuckoo search algorithm, and genetic algorithm, are also discussed. Obviously, in the current manufacturing industry, soft-sensing/soft sensors are hot subjects where a lot of research and development operations are ongoing. Most of the existing or ongoing work is confined to simulation-based experiments and only a few efforts have been accounted using soft sensors in industrial applications. Therefore, for the future advancement of the production industry, it is really essential to expand the research and development to develop soft sensors on the basis of industrial compatibility. The utilities of physical or hardware sensors cannot be undermined as these will be employed in information gathering for the functionality of the soft-sensing methods as well. Because of the prevailing circumstances, it can be estimated that in the future the world would be more inclined towards adjustable, authentic, adaptive, efficient and modern methods.

## Figures and Tables

**Table 1:** The performance difference between PSO-LS-SVM and LS-SVM.

Key Variables	LS-SVM		PSO-LS-SVM	
	RMSE	MAE	RMSE	MAE
**X**	0.2114	0.139	0.1028	0.067
**S**	1.2761	0.741	0.2597	0.125
**P**	0.0339	0.022	0.0160	0.008

X: cell concentration, S: substrate concentration, P: product concentration

**Table 2:** Comprehensive analysis of soft sensor models based on SVM (for the list of abbreviations see Table A1).

Ref	Prediction model	Compared with	RMSE/MSE	MAXE	Best Performance
[57]	**LS-SVM**	RBF-NN	-	-	LS-SVM
[45]	**LS-SVM**	NN	-	-	LS-SVM
[49]	**SVM**	BP-NN	-	-	SVM
[50]	**PSO-LS-SVM**		0.7835	0.486	PSO-LS-SVM
		LS-SVM	**0.1032**	**1.493**	
[51]	**PSO-SVM**	SVM	-	-	PSO-SVM
[52]	**ABC-MLS-SVM**	PID control	-	-	ABC-MLS-SVM
[54]	**GRA-LS-SVM**		2.518	2.641	GRA-LS-SVM
		RBF-NN	**14.273**	**6.271**	
		LS-SVM	**3.219**	**2.847**	
		GRA-RBFNN	**4.162**	**3.594**	
[56]	**IPSO-SVM**	PSO	-	-	IPSO-SVM
[58]	**MLS-SVM Inversion**	LS-SVM	-	-	MLS-SVM Inversion

Ref - References, RMSE – Root Mean Square Error, MAXE –Maximum Absolute Error, MSE – Mean Square Error

**Table 3:** Comprehensive analysis of soft sensor models based on NN/ANN (for the list of abbreviations see Table A1).

Ref	Prediction model	Compared with	RMSE/MSE	MAXE	Best Performance
[31]	NN-MIV		**27.512**	-	NN-MIV
		NN	37.943		
[61]	GPR-NN MIV		**0.0436**	**0.1440**	GPR-NN-MIV
		GPR	0.1082	0.4373	
[23]	RBF-NN	BP	-	-	RBF-NN
[62]	PSO-NN	BPNN	-	-	PSO-NN
[65]	Novel SS ANN	General SS-ANN	-	-	Novel SS-ANN
[66]	GRNN	RBF-NN	-	-	GRNN
[67]	KPCA-RBF-NN	PCA	-	-	KPCA-RBF-NN

Ref - References, RMSE – Root Mean Square Error, MAXE –Maximum Absolute Error, MSE – Mean Square Error.

**Table 4:** Comprehensive analysis of soft sensor models based on fuzzy logic (FL) NN/SVM (for the list of abbreviations see Table A1).

Ref	Prediction Model	Compared with	RMSE/MSE	MAXE	Best Performance
[43]	**FNN Inverse System**	PID control	-	-	FNN Inverse sys
[89]	**GD-FNN**		**0.0064**		
		RBF-NN	0.0194		GD-FNN
[90]	**MNN-KFCM**		**0.1963**	-	MNN-MFKC
		Single NN	0.5441		
[91]	**FLS-SVM**	LS-SVM	-	-	FLS-SVM
[92]	**PSO-FNN**		**0.1141**	**0.6151**	PSO-FNN
		FNN	1.3658	3.5640	
[93]	**Fuzzy LS-SVM**		**0.0097**	-	Fuzzy LS-SVM
		LS-SVM	0.0244		

Ref - References, RMSE – Root Mean Square Error, MAXE –Maximum Absolute Error, MSE – Mean Square Error.

**Table 5:** Comprehensive analysis of soft sensor models based on genetic algorithms (for the list of abbreviations see Table A1).

Ref	Prediction Model	Compared with	RMSE/MSE	MAXE	Best Performance
[96]	**GA-SVR**		**0.1353**	**0.8872**	GA-SVR
		ANN	0.9217	2.3529	
[97]	**GA-SVM**	NN	-	-	GA-SVM
	**GA-BPNN**	-	-	-	GA-BPNN
[98]	**Hybrid GA-ACO**	Conventional GA and stand-alone ACO	-	-	Hybrid GA-ACO

Ref - References, RMSE – Root Mean Square Error, MAXE –Maximum Absolute Error, MSE – Mean Square Error.

## Appendix A

**Table A1:** List of abbreviations.

Abbreviation	Explanation
**Methods**	
SVM	Support Vector Machines
SVR	Support Vector Regression
LS-SVM	Least Square Support Vector Machine
PLS	Partial Least Squares
MLS-SVM	Multiple Output Least Squares Support Vector Machine
MSE	Mean Square Error
RMSE	Root Mean Square Error
NN	Neural Network
DNN	Deep Neural Network
ANN	Artificial Neural Network
DL	Deep Learning
NLP	Natural Language Processing
DAE	Denoising auto-encoders
SAE	Stacking auto-encoders
HELM	Hierarchical Extreme Learning Machine
MIV	Mean Impact Value
BPNN	Back-propagation Neural Network
RBF	Radial Basis Function
GPC	Generalized Predictive Control
PID	Proportional-Integral-Derivative
GRA	Gray Relational Analysis
PSO	Particle Swarm Optimization
IPSO	Improved Particle Swarm Optimization
ABC	Artificial Bee Colony
ACO	Ant colony optimization
CS	Cuckoo Search
GA	Genetic Algorithm
KNN	K-nearest Neighbors
GPR	Gaussian Process Regression
GRNN	Generalized Regression Neural Network
KPCA	Kernel Principal Component Analysis
PCA	Principal Component Analysis
ICA	Independent Component Analysis
FL	Fuzzy Logic
FNN	Fuzzy Neural Network
GD-FNN	Generalized Dynamic Fuzzy Neural Network
MNN	Multi-model Neural Network
KFCM	Kernel Fuzzy c-means Clustering
FLS	Fuzzy Least Square
FCM	Fuzzy c-means Clustering
AIC	Akaike information criterion
PLVM	Probabilistic Latent Variable Models
RVM	Relevance vector machine
GMR	Gaussian Mixture Regression
JIT	Just-In-Time
PCR	Principal Component Regression
AHP	Analytic Hierarchy Process
